# Independency of Coding for Affective Similarities and for Word Co-occurrences in Temporal Perisylvian Neocortex

**DOI:** 10.1162/nol_a_00095

**Published:** 2023-04-11

**Authors:** Antonietta Gabriella Liuzzi, Karen Meersmans, Gerrit Storms, Simon De Deyne, Patrick Dupont, Rik Vandenberghe

**Affiliations:** Laboratory for Cognitive Neurology, Department of Neurosciences, Leuven Brain Institute, KU Leuven, Leuven, Belgium; Laboratory of Experimental Psychology, KU Leuven, Leuven, Belgium; Computational Cognitive Science Lab, University of Melbourne, Melbourne, Australia; Neurology Department, University Hospitals Leuven, Leuven, Belgium

**Keywords:** fMRI, representational similarity analysis, semantics, word embedding models

## Abstract

Word valence is one of the principal dimensions in the organization of word meaning. Co-occurrence-based similarities calculated by predictive natural language processing models are relatively poor at representing affective content, but very powerful in their own way. Here, we determined how these two canonical but distinct ways of representing word meaning relate to each other in the human brain both functionally and neuroanatomically. We re-analysed an fMRI study of word valence. A co-occurrence-based model was used and the correlation with the similarity of brain activity patterns was compared to that of affective similarities. The correlation between affective and co-occurrence-based similarities was low (*r* = 0.065), confirming that affect was captured poorly by co-occurrence modelling. In a whole-brain representational similarity analysis, word embedding similarities correlated significantly with the similarity between activity patterns in a region confined to the superior temporal sulcus to the left, and to a lesser degree to the right. Affective word similarities correlated with the similarity in activity patterns in this same region, confirming previous findings. The affective similarity effect extended more widely beyond the superior temporal cortex than the effect of co-occurrence-based similarities did. The effect of co-occurrence-based similarities remained unaltered after partialling out the effect of affective similarities (and vice versa). To conclude, different aspects of word meaning, derived from affective judgements or from word co-occurrences, are represented in superior temporal language cortex in a neuroanatomically overlapping but functionally independent manner.

## INTRODUCTION

Over the past decade, advances in computational modelling of word meaning have enabled novel ways of studying the representation of meaning in the human brain. Models of word meaning have relied on different types of sources. Data can be acquired from study participants performing explicit tasks, such as free word association (De Deyne et al., 2019), feature generation (De Deyne et al., 2008; McRae et al., 2005), or valence ratings (Moors et al., 2013; Van Rensbergen et al., 2016). According to such studies, word valence, or how positive/negative a word is, is one of the principal dimensions of the organisation of word meaning (Belyk et al., 2017; De Deyne & Storms, 2008; Kotz & Paulmann, 2011; Kousta et al., 2011; Kuperman et al., 2014; Meersmans et al., 2020; Osgood et al., 1957; Pauligk et al., 2019; Troche et al., 2017; Van Rensbergen et al., 2015; Vigliocco et al., 2014). Word valence is also highly relevant for how brain patterns are organised in response to words (Belyk et al., 2017; Meersmans et al., 2020, 2022; Pauligk et al., 2019). The effect of affective similarity (modelled as the Euclidean distance between words in a three-dimensional space formed by valence, dominance, and arousal ratings) is strong in the perisylvian language network and beyond (Meersmans et al., 2020, 2022).

As another highly influential approach to the representation of word meaning, word meaning models can make use of the distributional structure (Harris, 1954) present in the vast amount of pre-existing language corpora. Words can then be represented as word embeddings, i.e., continuous, low-dimensional, real-value vectors based on co-occurrences (Devereux et al., 2010; Hollis & Westbury, 2016; Mitchell et al., 2008; Pereira et al., 2013). Natural language processing (NLP; Abnar et al., 2018; Grave et al., 2019; Honnibal, 2017) is then applied to extract and detect regularities and statistical patterns of co-occurrence and transforms real-world language into computer-friendly real-valued vectors representations. A limitation, however, is that word embedding models have a rather limited ability to model the affective content of words, such as word polarity or other aspects of text sentiment (De Deyne et al., 2021; Tang et al., 2016; Yu et al., 2018). As these models are strongly context dependent, words with opposite polarity (e.g., “bad” vs. “good”) but similar contexts may be mapped near to each other by such models (Agrawal et al., 2018). Valence, arousal, and dominance can be derived from co-occurrence-based modelling but only provided that seed words are present of which the valence has been determined beforehand based on subjective ratings or lexica (“mots germes”; Recchia & Louwerse, 2015; Vincze & Bestgen, 2011). Even then, models based on task-generated data outperform co-occurrence-based models in predicting valence, arousal and dominance (Vankrunkelsven et al., 2018).

Hence, two of the principal ways of modeling word content, based on affective judgements versus word co-occurrences, capture different aspects of word meaning. The interest of this special relationship between co-occurrence-based modelling and affective word content can be gauged based on the number of high-quality prior studies from the NLP community (Tang et al., 2016; Yu et al., 2018), from computational linguistics (Westbury et al., 2015), psycholinguistics (Bestgen & Vincze, 2012; De Deyne et al., 2021), and experimental psychology on this topic (Hollis & Westbury, 2016; Recchia & Louwerse, 2015; Vankrunkelsven et al., 2018; Vincze & Bestgen, 2011; Westbury et al., 2015).

We examined how brain activity patterns relate to these two ways of representing word meaning that have been proven to be powerful in their own right but nevertheless appear to be fundamentally distinct. Given the distinctive nature of these representations, one might predict that they would rely on separate brain regions. Affective similarities have been reported to correlate strongly with the similarity of brain activity patterns in superior temporal language cortex (Meersmans et al., 2020), whereas co-occurrence-based similarities have been associated with a neuroanatomically relatively diffuse effect (Mitchell et al., 2008).

Word co-occurrences are influenced by word associations. Word associations can also be derived from other sources, such as experimentally using cued word generation (De Deyne et al., 2019; Vankrunkelsven et al., 2018). Hence, cued-association-based similarities hold a position in between co-occurrence-based and affective similarities. To gain further insight into the mechanism behind the correlations between co-occurrence-based similarities and brain activity patterns, we evaluated whether effects were comparable when similarities were based on task-generated word association data. This allows us to determine whether the critical factor determining the co-occurrence-based word similarity effects are the predictive computational modelling algorithms behind the word similarities or the word associations as such.

We re-analysed a functional magnetic resonance imaging (fMRI) data set from a study originally designed to investigate the representation of affective similarity between nouns. Nouns were presented in both auditory and in visual modality to further increase generalisability of our findings and analyses were pooled across modalities and tasks. Affective similarities were calculated from estimates of word valence, arousal and dominance, which were extrapolated from a data set of behavioural affective norms (Moors et al., 2013). These extrapolations were made based on a graph derived from a huge data set based on free word association (Van Rensbergen et al., 2016; for further details see Materials and Methods). Secondly, for the noun stimulus set used in this experiment, we re-calculated word similarities, using the word2vec vectors provided through the spaCy Python library, and examined how the corresponding brain activity patterns related to those obtained for the affective similarities. The primary word embeddings are based on the continuous bag of words (cBOW) approach. In this architecture, the target word is predicted based on the embeddings of its immediate context (e.g., 3–4 neighbouring words, depending on the window size) derived from a large internet-based text corpus, composed of Wikipedia entries and an extensive Webcrawl (>13 billion words; Honnibal, 2017). This study was meant to uncover commonalities and differences in coding mechanisms for similarities based on word associations/co-occurrences versus affective judgements, respectively.

## MATERIALS AND METHODS

### Subjects

Twenty-two subjects (15 women and 7 men, mean age 21.9, 19–24 years of age, all neurologically healthy and right-handed) participated. This sample size is in accordance with previous fMRI studies using the same approach and yielding replicable results (Bruffaerts et al., 2013; Carota et al., 2021; Liuzzi et al., 2015, 2019; Pauligk et al., 2019). All subjects were free of significant neurological or psychiatric history. Subjects provided informed consent before participating, and the experiments were approved by the Ethics Committee Clinical Studies UZ/KU Leuven.

### Stimuli

Stimulus words were selected from the 12,400 cue words studied as part of the Dutch Small World of Words (SWOW) data set (De Deyne et al., 2019).

**Figure 1.**
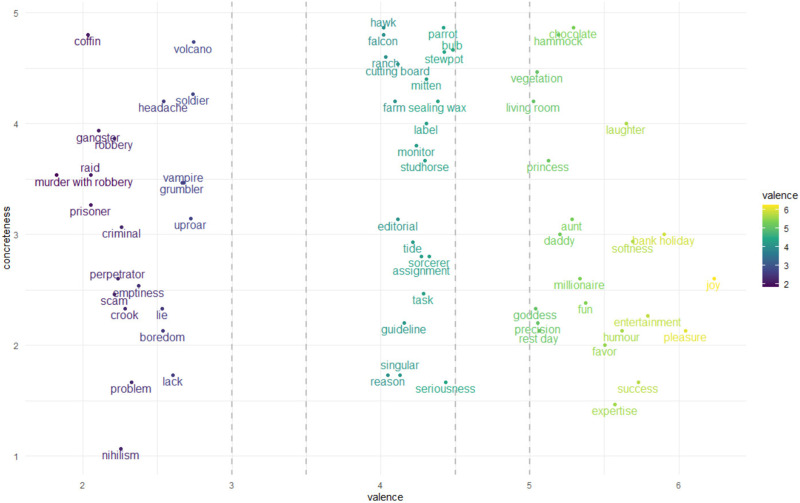
Stimulus set. The two coordinates of each noun correspond to valence (*x*-axis) and concreteness (*y*-axis).

A total of 66 nouns were distributed evenly over three classes: positive, neutral, and negative valence (Figure 1). Words were selected in a semiautomated manner based on the following criteria: (1) to maximise the range in semantic similarity, that is, contain word pairs that range between highly similar and highly dissimilar; and (2) to match words between the three valence classes for all relevant linguistic variables. The three groups were matched on age of acquisition, concreteness, dominance, log_10_(frequency), orthographic neighbourhood density, prevalence, and word length (Brysbaert et al., 2014; Keuleers et al., 2010, 2015; Marian et al., 2012; Van Rensbergen et al., 2016). The word selection script did not converge to a solution when arousal also needed to be matched between valence classes. Arousal was numerically lower for the neutral valence (mean = 3.98, *SD* = 0.49) class than for the positive (mean = 4.23, *SD* = 0.79) or negative valence class (mean = 4.54, *SD* = 1.05) (one-way analysis of variance: *F*(2, 63) = 2.62, *p* = 0.08). This is a consequence of the well-established U-shaped relationship between valence and arousal (Warriner et al., 2013).

The stimulus set is provided in Figure 1 and in Table S1 in the Supporting Information. The stimulus selection procedure yielded both concrete and abstract words without evident taxonomic structure (as measured based on WordNet; Miller, 1995).

### Experimental Design

The experiment was originally designed for the purpose of studying the effect of valence on fMRI activity patterns. The experiment had a 3 × 2 × 2 factorial design with stimulus type (positive, neutral, negative), modality (visual or auditory presentation), and overt articulation (yes/no) as factors. After a warning dot (500 ms), stimulus words were presented in visual or auditory modality (1,500 ms), followed by the presentation of a red or green dot (2,500 ms). Presentation of a green dot indicated to the subjects that they had to repeat the word aloud after stimulus presentation had ended. The red dot indicated that subjects had to remain silent. Visual stimuli were presented in white on a black background. Auditory stimuli were presented through OptoActive II active noise cancelling headphones (Optoacoustics, 2022) to minimise interference of scanner noise. The task was chosen to monitor subject engagement in the scanner, while limiting the effect of attentional orienting and control that can occur during explicit semantic tasks. To limit head motion, subjects were explicitly instructed to pronounce the words while restricting mouth movement to a minimum. Vocal responses were recorded using the OptoActive II FOMRI III microphone (Optoacoustics, 2022), which enables recording of responses in the fMRI environment in a sensitive manner. Head motion parameters were determined during image analysis, and runs were rejected based on a prior criterion (see below).

**Figure 2.**
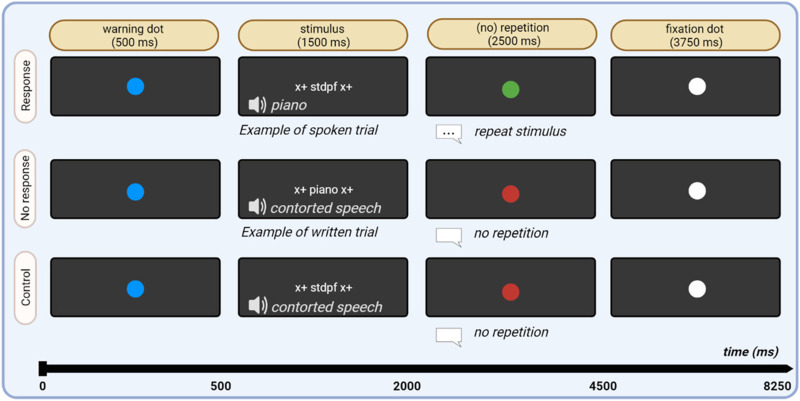
Experimental design.

The initial 1,500 ms phase following word onset was identical between covert and overt trials up to response cue onset. The covert and overt trials will be pooled in the current report. Following the response phase, a white fixation dot was presented until the start of the next trial. The interstimulus interval was 8.25 s. Every noun was presented once during each of the eight runs (4× in visual and 4× in auditory modality; duration per run: 10.5 min). Visual stimuli were accompanied by an auditory control (i.e., rotated spectrogram speech), while auditory stimuli were accompanied by a visual control (i.e., consonant letter string). Audio controls were generated from the stimuli by rotating the spectrogram around 50% of the maximal frequency and applying a low-pass filter at 95% of the maximal frequency. This procedure maintains certain spectral and phonetic features and intonation, while at the same time rendering the word unintelligible (Scott et al., 2000). Visual controls were generated by replacing the vowels in the original stimulus words by consonants and shuffling the letter order. During the control trials, the visual and auditory control stimuli were presented simultaneously, and no response was required (Figure 2).

Every run consisted of 77 trials (66 word trials + 11 control trials). The control trials were included as event type in the general linear model but were not used further in the current analyses. Every subject completed eight runs over two scan sessions. The word sets were presented in a pseudorandomised order and divided in a counterbalanced manner between the two sessions. This resulted in a total of eight replications per word (4 spoken and 4 written modality).

#### Word similarity matrices

**Figure 3.**
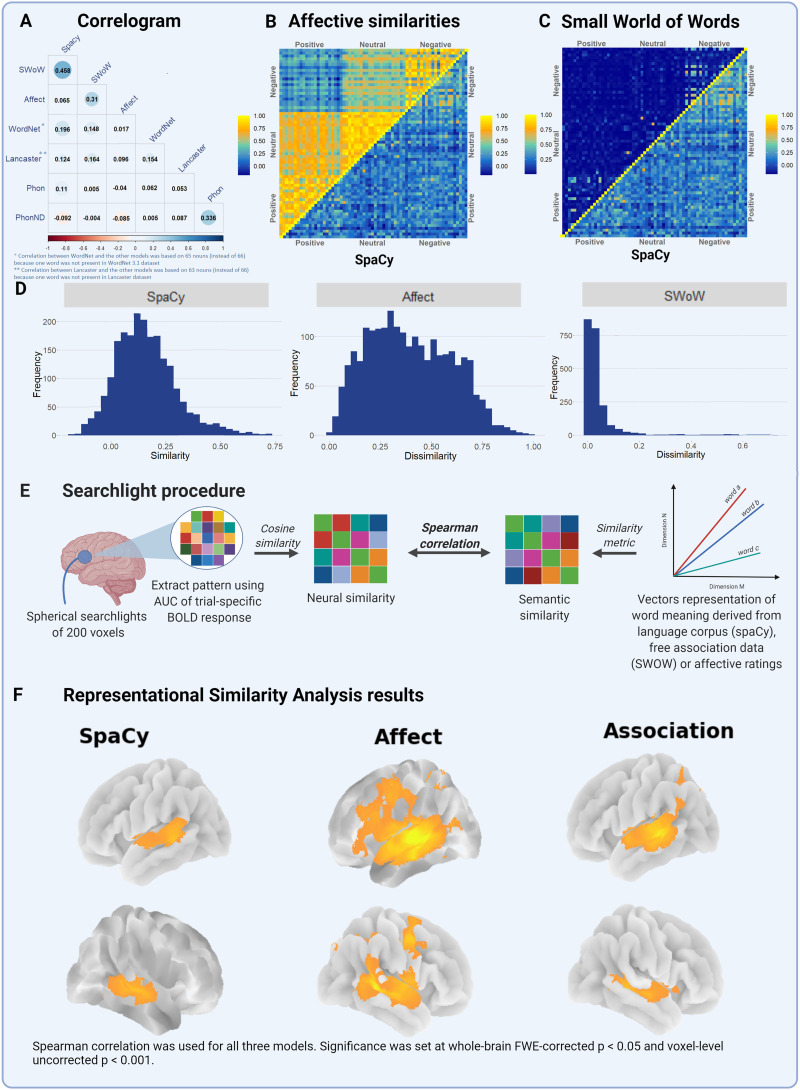
Overview of the word embedding models, the whole-brain representational similarity analysis (RSA) procedure, and the results. (A) Correlations between the different similarity matrices. (B) Affective similarity matrix (upper half of the matrix) next to the distributional similarity matrix derived from spaCy (lower half of the matrix). (C) Compares the Small World of Words and spaCy matrices in the same manner. (D) Distribution of the similarities from spaCy, affective ratings and Small World of Words (SWoW). (E) Provides a schematic overview of the whole-brain RSA using a 200 voxel searchlight. (F) Results of the RSA for all three models.

The primary objective of the current analysis was to examine how similarities derived from word co-occurrence-based models relate to similarities between fMRI activity patterns and to identify the commonalities and differences of these effects with those obtained for affective similarities. The word co-occurrence model was derived directly from spaCy (Honnibal, 2017). SpaCy is an open-source Python library for NLP. It contains built-in word embeddings (i.e., multidimensional vector representations of word meaning). Here, we use the word2vec word embeddings from spaCy’s large Dutch model (https://spacy.io/models/nl#nl_core_news_lg), which uses a cBOW approach on a combined corpus (web crawled text + Wikipedia). SpaCy similarities were normally distributed (Figure 3D; Figure S1 in the Supporting Information). The resulting similarity matrix was used for representational similarity analysis (RSA).

In the affective similarity model, we used valence, dominance, and arousal ratings (Van Rensbergen et al., 2016) to create a three-dimensional space. The pairwise Euclidean distance *d* between stimulus words was calculated, normalised, and converted to a similarity metric by 1 − *d*. Valence, dominance, and arousal ratings were taken from a large data set of affective estimates (Van Rensbergen et al., 2016). In this study, word valence, dominance, and arousal were extrapolated for 14,000 words from a smaller data set of behavioural norms (*n* = 4,300 words; Moors et al., 2013) using a *k*-nearest neighbour approach. The nearest neighbours were determined based on word embedding similarities derived from a free association graph (De Deyne et al., 2019). This graph is created from a large data set of continued associations (>12,000 words; see below for more details) with the words as nodes in the graph and the edges weighted by the association strength *p*(*response*|*cue*). In the affective ratings data set by Moors et al. (2013), word valence, dominance, and arousal were rated on a 1–7 scale. Only words for which these extrapolations were available were eligible for this study.

In a secondary analysis, for comparison with the spaCy based model, we included a behavioural similarity model derived from the Small World of Words word association data set (De Deyne et al., 2019). In this similarity model, word embeddings were derived from a large-scale continued association task, during which subjects were instructed to provide three associates for every cue (De Deyne et al., 2019). The SWOW data set comprises responses for over 12,000 cue words from 70,000+ subjects. From this data set, a graph is constructed using associative strength (i.e., probability of the response given a cue) as edge weight. Word embeddings were extracted from the graph using a random walk algorithm, and semantic similarity was calculated using cosine similarity (De Deyne et al., 2019; Liuzzi et al., 2019; Meersmans et al., 2020). Valence (pleasantness; positive [*holiday*] − negative [*murder*], dominance (power; strong [*avalanche*] − weak [*sleep*]), and arousal (activity; active [*explosion*] − passive [*silence*]) are important dimensions in this graph (Van Rensbergen et al., 2015). Furthermore, a direct comparison between word embeddings and word association indicated that the latter is better suited to capture affective information (De Deyne et al., 2021) The SWOW similarity matrix was not normally distributed (Figure 3D and Figure S1 in the Supporting Information).

To evaluate and control for the possible contribution of phonological similarities to the effects obtained, we calculated a phonological similarity matrix as 1 − the Levenshtein distance between phonological transcriptions of the stimuli. As a separate phonological model, we also calculated phonological neighbourhood density similarities as the difference in phonological neighbourhood density between stimuli (i.e., the number of words that can be constructed by substitution, addition, or deletion of one phoneme). Phonological transcriptions and neighbourhood density estimates were retrieved from the CLEARPOND database for Dutch (Marian et al., 2012; https://clearpond.northwestern.edu/dutchpond.php). These models were included to evaluate whether the observed semantic effects are in part driven by phonology.

Since prior studies have emphasised the role of taxonomic structure and of experiential similarities in the organization of brain activity pattern (Fernandino et al., 2022), we also calculated word similarity based on taxonomy and word similarity derived from experiential judgements for our set of nouns and tested the correlation with the dimensions of primary interest, the affective similarities, and the co-occurrence-based similarities. Taxonomic structure was modelled based on WordNet (https://wordnet.princeton.edu/). WordNet similarity matrix was calculated based on Wu and Palmer (1994) similarity (WPsim). WPsim calculates the relatedness between two words by considering the depths of the two synonyms (i.e., synsets) in the WordNet taxonomies, along with the depth of the least common subsumer (i.e., the most specific common hypernym). Experiential strength was based on the Lancaster sensorimotor norms (Lynott et al., 2020). Lancaster similarity matrix was calculated by computing the cosine similarity for all pairs of concept vectors (see Table 1 for an overview of the models). The taxonomic and experiential similarities were calculated from English translations of the Dutch nouns. Three nouns were absent from the Lancaster norms and one noun was absent from WordNet, hence these were left out (Table S1, for Dutch nouns and English translations).

**Table 1.** Overview of the models.

	Type	Data	N	Procedure	Similarity
SpaCy	Co-occurrence	Wikipedia and webcrawl	300	cBOW	Cosine
Affective similarties	Task-based	Valence, dominance, and arousal ratings	3	Human ratings extrapolated based on SWOW similarities	Euclidean
Small World of Words	Task-based	Semantic associations	NA	Random walk	Cosine
Phonological similarities	Phonological	Transcriptions from CLEARPOND	NA	Number of insertions, deletions or substitutions needed to change one string into the other	Levenshtein
Phonological neighbourhood density	Phonological	Total number of words that differed in the addition, deletion, or substitution of a single phoneme	1	NA	Absolute value of the difference in phonological neighbourhood density
Taxonomic structure	Taxonomy	WordNet v31	NA	Knowledge graph where words are grouped into synsets	Wu & Palmer similarity
Experiential strength	Task-based	Lancaster sensorimotor norms	12	Human ratings for 39,707 concepts across six perceptual modalities and five action effectors	Cosine similarity

*Note*. *N*: number of dimensions in the model. cBOW: continuous bag of words. SWOW: Small World of Words.

#### Correlations between similarities from the different models

Correlations between the different word embedding models show the difference in overlap between models (Figure 3A). The correlations between the co-occurrence model and the association-based model was 0.458, showing the partial collinearity between the models. This is in line with the view that co-occurrence-based models capture some of the meaning encoded in word associations.

In contrast, the affective similarity model correlated poorly with the co-occurrence-based model (*r* = 0.065). Affective similarity was better captured by the association-based model (*r* = 0.310) than by the co-occurrence-based models. This difference was formally evaluated using the cocor package for R (Diedenhofen & Musch, 2015), indicating that the correlation between the affective and association-based model was significantly stronger than the correlation between the affective and co-occurrence-based model (Pearson and Filon’s *z* = −11.4; *p* < 0.0001).

The correlations between the phonological (neighbourhood density) matrix and the semantic matrices were overall very low (Figure 3A). The Spearman correlation of the co-occurrence-based similarities with the taxonomy-based similarities was 0.196, and with the experiential strength-based similarities 0.124. The Spearman correlation of the affective similarities with the taxonomy-based similarities was 0.017, and with the experiential-strength-based similarities 0.096. Despite the low or negative correlations with phonological distance and neighbourhood density, taxonomy-based or experiential-strength-based similarity matrices, we also calculated the RSA effect of co-occurrence-based and affective similarities after partialling out each of these factors.

### Image Acquisition

**Figure 4.**
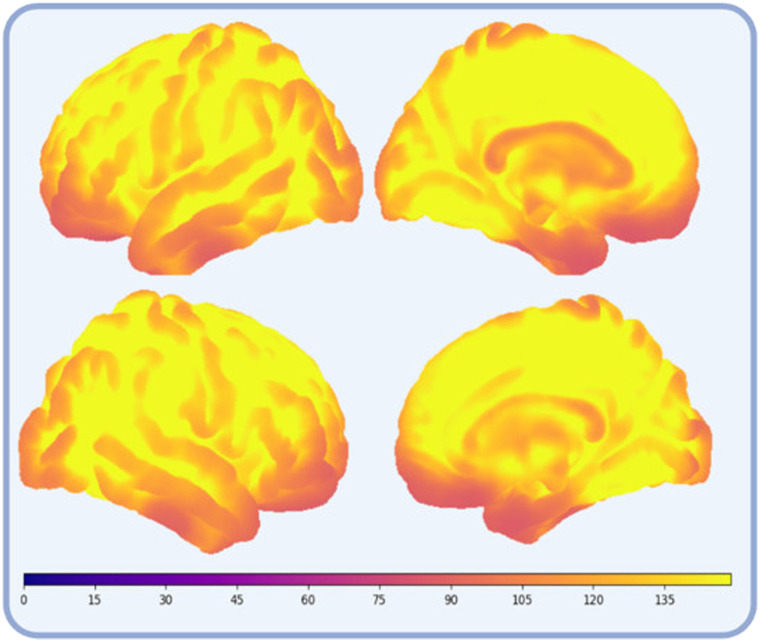
Temporal signal-to-noise ratio, calculated by dividing the mean of the residual time series by its standard deviation.

Functional and structural fMRI images were acquired on a 3T Philips Achieva with a 32-channel head coil and equipped with the OptoActive II ANC headphones and FOMRI III microphone. Functional images were acquired using a T2* sequence with 36 slices (multiband acceleration factor = 2; repetition time = 1 s; echo time = 30 ms, field of view = 220 × 135 × 220 mm^3^, voxel size = 2.75 × 2.75 × 3.75 mm^3^). The temporal signal-to-noise ratio was calculated by dividing the mean of the residual time series by its standard deviation (Murphy et al., 2007; Figure 4). Structural images were obtained using a T1- weighted 3D turbo-field-echo (repetition time = 9.6 ms, echo time = 4.6 ms, in-plane resolution = 0.97 mm, slice thickness = 1.2 mm) in every subject.

### Image Preprocessing

Images were submitted to a preprocessing pipeline in SPM12 (Ashburner et al., 2021). Functional images were realigned to correct for head motion, followed by slice timing correction. Next, functional and structural images were coregistered and the anatomical images were segmented into white matter, grey matter, and cerebrospinal fluid. The grey matter probability maps are used to restrict the analysis to cortical voxels. The anatomical and functional images were normalised to Montreal Neurological Institute (MNI) space. No smoothing was applied to suit the multivariate nature of our analyses. During realignment, motion parameters were generated. Runs where the framewise displacement (the sum of the absolute values of the differentiated realignment parameters) exceeded 1 mm were excluded from further analysis.

### Statistical Analysis

We applied RSA in a whole-brain searchlight procedure using the CosMoMVPA toolbox (Oosterhof et al., 2016). Trials were pooled over tasks (overt articulation and covert trials), over word classes (positive, neutral, negative) and over modalities (auditory and visual). Control trials where no words were presented were not included. Figure 3E provides an overview of the searchlight procedure.

Trial-specific activation maps were used as input for this analysis. These maps were generated by extracting the voxelwise blood oxygen level dependence (BOLD) response from the normalised, unsmoothed images and calculating the area under the curve of the BOLD response in every voxel from 2 to 8 s. This procedure is identical to that applied in previous multivariate pattern analysis (MVPA) studies (Bruffaerts et al., 2013; Liuzzi et al., 2015, 2017; Meersmans et al., 2020, 2022). The number of voxels per searchlight was set a priori to 200 (corresponding to a sphere radius = 11 mm; voxel size equal to 2 × 2 × 2 mm^3^) and the recommended data centring was applied before the analysis. The searchlight analysis was limited to subject-specific grey matter masks, created by thresholding the grey matter probability maps at 0.3. In every searchlight, the activity pattern was extracted from the activation maps and pairwise similarities were estimated using cosine similarity. The search sphere in CosMoMVPA may contain voxels from opposite walls of a sulcus. During the analysis, subject-specific voxelwise RSA maps were created containing the correlations between the activity similarities of the searchlight centred on a specific voxel and the word similarity model. The distribution of the task-based similarities (affective matrix and free association-based matrix) deviated from normality (Figure 3D; Figure S1). Therefore, Spearman correlation was used in the searchlight RSA for all models to facilitate between-model comparison. These maps were *r* to *z* transformed and smoothed before being submitted to a group-level *t* test using SPM12. At every voxel, the *t* test evaluates whether the correlation is significantly different from zero across subjects. These *t* tests were performed for every searchlight RSA separately. To find significant differences between pairs of word similarity models, the same maps were submitted to a paired *t* test. Significance at the group level was set to a whole-brain familywise error (FWE)-corrected threshold of *p* < 0.05 (with uncorrected voxelwise *p* < 0.001).

Finally, in order to address the unique relation between a specific model (e.g., co-occurrence) and the neural representation, partial correlation searchlight RSAs were conducted. The procedure was identical to the one described above, with the only difference that the correlation between a model representational dissimilarity matrix (RDM; e.g., co-occurrence) and the neural RDM was controlled for another model (e.g., affective).

## RESULTS

On average, subjects made 3.7 (range 0–14) repetition errors out of 264 overt articulation trials (i.e., wrong word or no repetition) over the total of eight runs. Over all subjects, a total of eight runs (4.5%) had to be excluded due to excessive head (framewise displacement > 1 mm).

### Effect of Co-Occurrence-Based Similarities

Using searchlight RSA, similarities between words estimated using word2vec in spaCy correlated significantly with the similarity of activity patterns in the lateral temporal neocortex surrounding the superior temporal sulcus (STS) from the posterior end to the middle third, to the left and also to the right (Figure 3F; Table 2). Table 3 provides the Spearman correlation and 95% confidence interval (CI) of the local maxima yielded by the group-level (SPM12) analysis of the searchlight RSA for co-occurrence similarity. Since the analysis is based on a random-effects model, the Spearman correlations provided are the average of the Spearman correlations obtained in each of the individuals. No other regions showed a significant similarity effect.

**Table 2.** Results of the whole-brain searchlight representational similarity analysis (RSA) with word2vec similarities (spaCy) and affective similarities.

**RSA results for co-occurrence-based similarities**						
Label	Size	FWE *p*	Peak coordinates			*t*(21)
L posterior STS	782	<0.001	−57	−34	2	5.48
			−54	−13	−4	4.75
			−69	−1	−4	4.56
R posterior STS	1,023	<0.001	54	−37	5	5.37
			48	−28	5	5.33
			57	−22	−1	5.05
**RSA results for affective similarities**						
Label	Size	FWE *p*	Peak coordinates			*t*(21)
Left and rightSTS, left MTG, precentral gyrus, precuneus. Left middle and inferior FC, left FG	13,319	<0.001	−69	−25	5	8.60
			−51	−34	5	7.54
			−36	−64	−1	7.27
**RSA results for free association-based similarities**						
Label	Size	FWE *p*	Peak coordinates			*t*(21)
Left STS, left rostral AG, left FG	2,423	<0.001	−57	−34	−1	6.78
			−42	−43	−7	5.65
			−66	−1	−4	5.05
Right STS	1,210	<0.001	51	−37	−1	5.32
			54	−16	−1	5.17
			48	−28	5	5.12

*Note*. Spearman correlation was used for all analyses. Significance was set at a whole-brain FWE-corrected threshold of *p* < 0.05 at the cluster level (after applying an uncorrected voxelwise threshold *p* < 0.001). Size: cluster size in number of voxels (2 × 2 × 2 mm^3^), in MNI space. FWE *p*: FWE-corrected *p*-value at the cluster-level. Abbreviations: STS: superior temporal sulcus; MTG: middle temporal gyrus; FC: frontal cortex; FG: fusiform gyrus; AG: angular gyrus; FWE: familywise error.

**Table 3.** Correlational values and 95% confidence interval (CI) based on average correlation value across volunteers.

(A) RSA for co-occurrence similarity						(F) RSA for affective similarity					
Local maxima			Rho	Lower CI	Upper CI	Local maxima			Rho	Lower CI	Upper CI
−57	−34	5	0.026	−0.018	0.066	−69	−25	5	0.034	−0.007	0.076
(B) RSA for co-occurrence similarity corrected for affective similarity						(G) RSA for affective similarity corrected for co-occurrence similarity					
Local maxima			Rho	Lower CI	Upper CI	Local maxima			Rho	Lower CI	Upper CI
−57	−34	2	0.020	−0.021	0.063	−69	−25	5	0.034	−0.008	0.076
(C) RSA for co-occurrence similarity corrected for association–based similarity						(H) RSA for affective similarity corrected for association–based similarity					
Local maxima			Rho	Lower CI	Upper CI	Local maxima			Rho	Lower CI	Upper CI
Not significant						−69	−25	5	0.030	−0.012	0.072
(D) RSA for co-occurrence similarity corrected for taxonomic similarity (WordNet)						(I) RSA for affective similarity corrected for taxonomic similarity (WordNet)					
Local maxima			Rho	Lower CI	Upper CI	Local maxima			Rho	Lower CI	Upper CI
−57	−34	2	0.026	−0.016	0.068	−69	−25	5	0.033	−0.009	0.075
(E) RSA for co-occurrence similarity corrected for experiential similarity (Lancaster)						(J) RSA for affective similarity corrected for experiential similarity (Lancaster)					
Local maxima			Rho	Lower CI	Upper CI	Local maxima			Rho	Lower CI	Upper CI
−57	−34	2	0.021	−0.021	0.064	−69	−25	5	0.033	−0.008	0.076

*Note*. Values are reported for the left posterior temporal peak local maxima based on the group-level (SPM12) of the (A) RSA for co-occurrence similarity, RSA for co-occurrence similarity (B) corrected for affective similarity, (C) corrected for association-based similarity (D) corrected for taxonomic similarity, (E) corrected for experiential similarity. (F) RSA for affective similarity, RSA for affective similarity (G) corrected for co-occurrence similarity, (H) corrected for association-based similarity, (I) corrected for taxonomic similarity, (J) corrected for experiential similarity.

**Figure 5.**
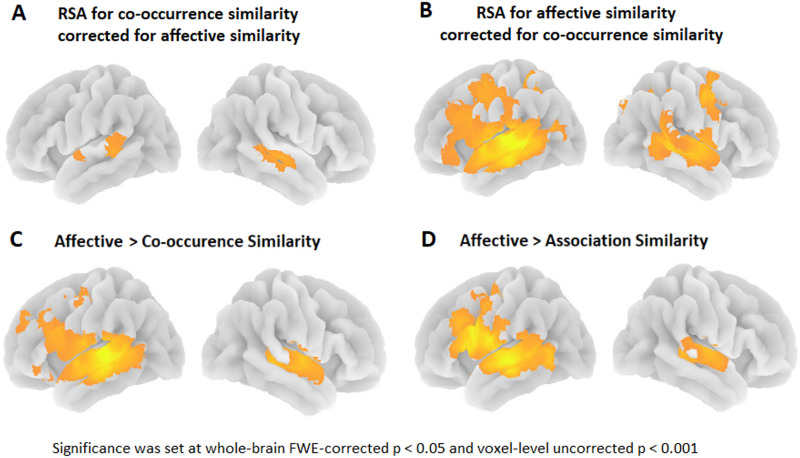
3D rendering of the (A) semantic similarity effect for co-occurrence similarity corrected for affective similarity; (B) semantic similarity effect for affective similarity corrected for co-occurrence similarity; (C) paired *t* test showing the correlation strength of affective similarity stronger than co-occurrence similarity; (D) paired *t* test showing the correlation strength of affective similarity stronger than association-based similarity. RSA: representational similarity analysis; FWE: familywise error.

We verified the similarity between the word2vec based matrix (spaCy) and the fMRI activity patterns after partialling out the effect of affective similarity and association-based similarities. The RSA results remained essentially the same when controlling for affective similarities (L pSTS peak coordinates: −57, −34, 2; *t*(21): 4.80; cluster size: 377 voxels; cluster level FWE-corr *p* = 0.002; R pSTS peak coordinates: 48, −28, 2; *t*(21): 4.83; cluster size: 702 voxels; cluster level FWE-corr *p* < 0.001; Figure 5A). This is logical given the very low correlation between the co-occurrence-based model and the affective similarity model. When controlling for the association-based similarities, no significant effects of the co-occurrence similarities were observed, in line with the moderate degree of collinearity between these two similarity models. As expected based on the correlogram between the different matrices (Figure 3A), the results remained unchanged when controlling for phonological similarity or similarity in phonological neighbourhood density using partial correlations (Table S2A and S2B). It also remained unchanged when controlling for taxonomy-based or for experiential similarities (Table S2C and S2D).

### Comparison to the Effect of Affective Similarities

For the affective similarities, strong effects were obtained in the lateral temporal perisylvian neocortex, and more distributed effects outside of lateral temporal cortex were also observed, namely in left middle and inferior frontal regions and fusiform gyrus (Figure 3F; Table 3F–J), which provides the Spearman correlation and 95% CI of the local maxima yielded by the group-level (SPM12) analysis of the searchlight RSA for affective similarity. Using a paired *t*-test across subjects, we evaluated significant differences between the models. Correlations were significantly higher for the affective similarities compared to spaCy- or association-derived similarities in bilateral superior and middle temporal gyrus and left middle and inferior frontal gyrus (paired *t* test; FWE-corrected cluster-level *p* < 0.05 combined with uncorrected voxel-level *p* < 0.001; Figure 5C and D). No regions were identified where spaCy- or association-derived similarities had a stronger effect than affective similarities. The effects of affective similarities remained unaltered when partialling out the effects of co-occurrence-based (peak-coordinates: −69, −25, 5; *t*(21) = 8.68; cluster size = 13,143 voxels; cluster level FWE-corr *p* < 0.001; Figure 5B) or association-based similarities. Similarly, results did not change after partialling out phonological distance, phonological neighbourhood density, taxonomy-based and experiential-based similarity (Table S3).

### Effect of Free Word Association-Based Similarities

Co-occurrence-based models are influenced by word associations. We also evaluated the effect of association-based similarities derived from SWOW. In a secondary analysis, we repeated the RSA using a behavioural similarity matrix derived from free association data (SWOW data set). The results of the SWOW similarity matrix overlapped with those for the word2vec matrix in STS but extended more posteriorly into the rostral angular gyrus and inferiorly into the fusiform gyrus (LH: 2,423 voxels, peak coordinate −57, −34, −1; RH: 1,210 voxels, peak coordinate 51, −37, −1; Table 2; Figure 3F).

## DISCUSSION

There was a striking correspondence between word co-occurrence-based similarities (word2vec) and similarities in activity patterns in the neocortex surrounding the left STS, and this was also true for affective similarities. Despite this co-localisation in superior temporal neocortex, the co-occurrence and affective similarity effects in lateral temporal neocortex were independent, in line with the low correlation between the two similarity matrices. In contrast, the results obtained with the co-occurrence-based and SWOW word association model showed a strong resemblance to each other, in line with the correlation between the word similarities derived from these two types of word association models.

### Comparison to Previous fMRI Studies Using Word2vec Modelling

The co-occurrence-based similarities were localised to a region confined to the lateral superior temporal cortex. This contrasts with some of the earlier studies of co-occurrence-based similarities showing a neuroanatomically distributed correlation between co-occurrence-based word similarities and the similarity of brain activity patterns. In a landmark study, Mitchell et al. (2008) described a predictive relation between co-occurrence data and neural activity. Each object stimulus word was represented as a 25-dimensional vector, with each value corresponding to the normalised sentence-wide co-occurrence of that word with 25 sensorimotor verbs (e.g., see, hear, eat; see also Anderson et al., 2013). While dimensionality reduction is an integral part of the word2vec approach applied here, the dimensions retained are entirely data driven based on the corpora analysed (300 dimensions in the spaCy model). The low-dimensional sensorimotor approach could potentially explain the high congruence between predicted and observed data in visual, motor, or gustatory cortex in the Mitchell et al. (2008) study. Representations in lateral temporal cortex seem to be less dependent on sensorimotor information, but instead rely on language-internal sources. These representations are also better modelled in a data-driven space with more dimensions than when dimensions are defined by the investigator. When the Mitchell et al. (2008) data set was analysed using word2vec, good concordance was achieved between the model and the overall brain activity pattern (Abnar et al., 2018). However, in that study the neuroanatomical network underlying this result was not the main topic of interest and relatively poorly defined.

Another study using co-occurrence-based vectors also reported a more distributed pattern than the focalised lateral temporal region we found (Pereira et al., 2018). Between-study differences in the similarity modelling can explain the difference in outcome. In Pereira et al. (2018) the co-occurrence-based vectors form the basis for spectral clustering that divides the semantic space in approximately 180 clusters that are then each represented by a representative word selected by the examiners. Next the authors derive dimensions from the set of representative words. During the fMRI experiment the words were embedded in sentences that also contained other words from the same cluster. In contrast to Pereira et al. (2018) the current paper directly imports the word embeddings as determined by spaCy, without further processing steps. It is conceivable that the sequence of meaning-oriented procedures in Pereira et al. (2018) may lead to a semantic representation that is richer in content than the representations based purely on corpus-derived word embeddings. This may then explain the wider neuroanatomical distribution of the effects in Pereira et al. (2018) compared to the current findings.

In an approach similar to the current one, Wang et al. (2018) performed a RSA with three similarity matrices: based on co-occurrences, based on semantic features, and based on subjective ratings. All words were abstract. Co-occurrence-based similarities were correlated with similarities of activity patterns in perisylvian language regions in temporal and frontal cortex (based on a volume of interest encompassing the perisylvian language network in its entirety), whereas the more semantically based similarities resulted in a more distributed pattern. In their globality, the findings by Wang et al. (2018) indicate that the perisylvian language network codes for co-occurrence-based similarities. The current findings confirm this observation in a whole-brain RSA in a larger group of participants (22 vs. 6), a wider range of words and input modalities, and without prior word selection step based on stability of responses. Another finding in common with Wang et al. (2018) as well as other studies (e.g., Meersmans et al., 2020) is the strength of the effect of affective similarities. As a major difference with Wang et al., in a whole-brain search analysis, the co-occurrence-based-similarities were limited to the cortex surrounding the STS in our study while they were found in ventral and middle premotor cortex in Wang et al. (2018) (mostly in the ventral and middle premotor cortex according to Figure 4B). Furthermore, the correlation with the co-occurrence-based model in the cortex surrounding the STS was bilateral in the current study and left lateralised in the study by Wang et al. (2018). The difference in localisation may be due to a variety of reasons, for instance, the use of a broader range of words in our study compared to abstract words only in Wang et al., and the use of auditory along with visual modality.

A fourth study (Fernandino et al., 2022) revealed that representational similarity for co-occurrence-based similarities was weaker than for taxonomy-based similarities or, even more so, similarities based on componential experiential semantics (Binder et al., 2016). For that reason, we repeated the RSA after partialling out taxonomic and experiential similarities. Results remained essentially the same. The difference between studies can be accounted for by the difference in composition of the stimulus set. In the current study, words were equally distributed between three valence classes and selected semiautomatically to maximise the range of the pairwise semantic similarities controlling for a list of relevant variables. The stimulus set did not contain an evident taxonomic structure and many of the experiential features of Binder et al. (2016) were not applicable to the words we used. The difference between studies demonstrates that RSA reflects the structure present in the word set. Comparisons between the effects of different dimensions within a word set are valid but should not be interpreted in absolute terms, and the outcome will depend on the structure present in the experimental set of words. Had we composed a word set where words were evenly distributed over the traditional semantic categories, it is likely that the effect of taxonomic structure would have been much more prominent (Fernandino et al., 2022).

### Comparison Between the Results for Co-Occurrence and Affective Similarity-Based Models

The lateral temporal region that showed co-occurrence-based similarities in our study also showed strong affective similarity effects. When the affective similarity effect was partialled out, the co-occurrence-based effect remained unchanged, as expected given the low correlation between the co-occurrence-based similarities and the affective similarities (Figure 3). Hence, the cortex surrounding the STS codes for co-occurrences and for affective similarities likely through relatively separate operational mechanisms.

Affective information is particularly difficult to capture from co-occurrence-based word embeddings (De Deyne et al., 2021; Tang et al., 2016; Yu et al., 2018): The correlation between affective and co-occurrences models equalled 0.065, while the correlation between affective and free word association-based models was 0.310. In the current study, the affective model resulted in stronger and notably more extensive effects than the co-occurrence model in the RSA. The effect of affective similarities was present in the STS, replicating previous findings (Meersmans et al., 2020), but was more widespread in line with the distributed representation of word meaning (Binder & Desai, 2011; Huth et al., 2016). The effect of co-occurrence-based similarities cannot be accounted for by the affective similarity effect, as it was unaltered when partialling out the affective similarity effect. Models that are derived from evaluative judgements by human subjects capture word meaning to a fuller extent than those that are derived from co-occurrences in text corpora, and this is a plausible explanation of the stronger and more extensive effects. However, the effect of co-occurrence-based similarities remain after partialling out the affective similarities, and this indicates that the two models represent different information about words and not that one is simply a weaker version of the other.

The comparison with SWOW-based similarities is of interest because they occupy a position somewhat in between co-occurrence-based similarities and affective similarities. SWOW is based on a cued free association task. It shares with affective similarities the origin in subjective judgements by human participants and with co-occurrence-based similarities its strong link with word associations. This intermediary position between co-occurrence-based and affective-judgement-based modelling is also evident from the correlogram (Figure 3A): SWOW-based similarities correlate both with co-occurrence-based similarities and with affective similarities. When SWOW word association similarities were partialled out, the co-occurrence-based effect was no longer significant. Co-occurrence and free word association-based similarities share a substantial amount of information (*r* = 0.458; Figure 3A), but association-based models are known to better capture affective information. With the current correlational approach, it is impossible to disentangle whether the co-occurrence-based or the word-association-based similarities are driving the effect more strongly given the collinearity between the two matrices (Figure 3A).

### Hypothetical Functional-Anatomical Model of Superior Temporal Cortex

Taken together, the findings from the co-occurrence-based and the affective model may be understood in the context of the theory of a hybrid conceptual system with symbolic and embodied representations (Andrews et al., 2009; Louwerse & Jeuniaux, 2010; Paivio, 1991). The co-occurrence-based model yielded strong effects specifically in the left STS, the affective similarity model yielded even stronger, but independent, effects in this region, and beyond. A hybrid model could explain why both affective similarity and word2vec similarities correlate significantly with the superior temporal neocortex, despite a relatively low correlation between the two similarity matrices (Figure 3A). The two processes, one co-occurrence/association-based and the other driven by valence as one of the principal dimensions of word meaning, may operate in a same region in a relatively independent manner.

The lateral temporal region to the left corresponds to what is also known as Wernicke’s area, although its exact anatomical boundaries are debated (Binder, 2015). Wernicke already hypothesised about the role of co-occurrences in the formation of associations between cortical responses (Gage & Hickok, 2005), a mechanism that has since been shown to be important in processing at distinct linguistic levels (Blank & Davis, 2016; Forseth et al., 2020; Schuster et al., 2021). The activated zone surrounding the STS has been previously implicated in human word intelligibility in a very consistent manner (Davis & Johnsrude, 2003; Evans et al., 2014; Obleser & Kotz, 2010; Scott et al., 2000). Word intelligibility requires integration of multiple, hierarchical levels, from the acoustic over the phonological to the semantic level (Obleser, 2014) and can be manipulated bottom up (e.g., by filtering the acoustic signal) or top down (e.g., predictability based on sentence context; Obleser & Kotz, 2010). Obviously, in connected speech, statistical regularities are extremely useful for predicting and recognizing words efficiently despite the high word rate and any degradation of the acoustic signal that may exist. Even for artificially isolated words, word identification may be guided by a scaffold of learnt co-occurrences. Here, the terms context and prediction do not refer to actual experimental context or predictions, but to probabilities learnt from language exposure. In connected speech, these co-occurrence-based regularities may be very informative for predictive coding (Arnal et al., 2011; Blank & Davis, 2016).

The role of superior temporal cortex in coding word associations/co-occurrences was relatively independent from its role in coding affective similarities. Estimating affective similarities requires subjective ratings and can be based on human ratings or on dictionaries and lexica of data, such as emoticon labels (Tang et al., 2016), labelled by experts. The current data confirm that these affective dimensions, known to be important for meaning representation for decades (Osgood et al., 1957), are also expressed in the perisylvian lateral temporal cortex (Meersmans et al., 2020). Intuitively one could have expected a more prominent role of so-called emotion processing regions rather than language regions for coding affective word similarities. However, the current findings confirm earlier and consistent observations of the coding of affective similarities by superior lateral temporal regions (Meersmans et al., 2020). Words with positive or negative valence are generally more abstract than neutral valence words. Abstract words more heavily rely on the linguistic system for their meaning than concrete words (Borghi & Binkofski, 2014; Kousta et al., 2011; Louwerse, 2011; Vigliocco et al., 2014). We have previously postulated that this may relate to the strong effect of affective similarities in superior temporal perisylvian language cortex (Meersmans et al., 2020). Neurobiologically, this is a correlate of the entwinement between affect and language (Wilce, 2009).

Our results do not imply that superior temporal cortex activity patterns are exclusively explained by either association-based similarities or affective similarities for any set of nouns. As mentioned before, results of RSA mirror the structure within the stimulus set employed. Activity patterns in superior temporal language cortex certainly code for other dimensions too, such as taxonomic structure or compenential experiential semantic structure (Fairhall & Caramazza, 2013; Fernandino et al., 2022). This by no means contradicts our findings. Which model yields the best correlations between word similarity and similarity in activity patterns depends on the type of stimulus set and maybe also the task performed (Meersmans et al., 2022). For a word set with a high proportion of concrete words and hence a clear taxonomic structure and strong sensory embodiment, an experiential model may be superior to a co-occurrence-based model (Fernandino et al., 2022). However such an observation should not be generalised to all types of word sets. The current stimulus set has been selected semiautomatically to maximise the range in pairwise similarities and to control for the proportion of positive, neutral, and negative valence stimuli. It was not generated in order to have a representative and consistent sample of traditional semantic categories distributed over the three valence classes. As a consequence, the current stimulus set did not contain a clear taxonomic structure and several of the 64 features of a prevailing componential experiential semantic model (Binder et al., 2016) were not applicable to the current stimulus set. It is important to put fMRI representational similarity results in the context of the type of word stimuli examined. As a consequence, exclusionary claims towards the representation of dimensions of word meaning in the lateral superior temporal language cortex are unwarranted. When the stimulus set contains a clear categorical structure, categorical effects can be found in superior temporal cortex (Carota et al., 2021; Devereux et al., 2013), and likewise for taxonomy-based structure (Fernandino et al., 2022) or experiential sensorimotor strengths (Carota et al., 2021; Fernandino et al., 2022). The structure within the experimental set of nouns will determine which dimensions are most strongly represented by superior temporal cortex in response to these nouns in a dynamic, context-dependent manner.

### Limitations

The use of overt articulation during fMRI can potentially introduce motion artefacts. The state-of-the-art voice recording system with noise cancellation enables the recording of responses in a sensitive manner even when speech volume is low. Subjects were specifically instructed to pronounce the words without moving their mouths excessively and without head motion. Head motion was evaluated and runs where the a priori threshold for framewise displacement (>1 mm) was exceeded were excluded from further analysis.

Trials with overt articulation and trials without overt responses were pooled for RSA. This increases the generalisability of our findings. The subjects received the task instruction only after the word stimulus had been shown so that the initial phase (1,500 ms duration) of conscious word processing is matched regardless of response or judgement. For repetition semantic processing is not necessary (Jefferies et al., 2006) although in the intact brain conscious word perception will inevitably activate a certain level of semantic processing. This is also evidenced by the empirical effects of affective and word association-based similarities upon the brain activity pattern.

The word sets were selected to be evenly distributed across different word valence classes. They may therefore not be representative for the total noun thesaurus which is not distributed evenly across negative, neutral, and positive valence classes. This could introduce a bias in favour of affective similarity modelling versus word co-occurrence-based modelling. However, the main finding is the independency of coding between the two types of similarities rather than the strength of the effect of one versus the other approach.

The affective ratings used to create the three-dimensional affective similarity space were extrapolated from a smaller set of behavioural ratings based on similarities derived from the SWOW association data set. As such, the stronger overlap between the association-based and affective models can be in part explained by this procedure. On the other hand, this underlines our central conclusion of independent coding of co-occurrence-based and affective similarities: Using extrapolations could potentially have introduced a similarity-based confound into the affective estimates.

Behaviourally, there was no correlation between the affective similarity matrix and the co-occurrence-based similarity. This observation fits with the initial rationale of the current experiment, namely that co-occurrence-based similarities capture affective similarities poorly and that for affective similarities to be calculated requires subjective input. The word set we chose was not specifically selected for an absence of correlation between affective and co-occurrence-based similarities, nevertheless for other word sets such a correlation may be found, although we would expect it in general to be relatively weak. The conclusion of functional independence of the superior temporal representation of affective and co-occurrence-based similarities is based on the absence of a behavioural correlation, the co-localisation as well as the absence of any effect of partialling out the effects of co-occurrence or affective similarities, respectively. The absence of an effect of partialling out the effects of co-occurrence or affective similarities follows mostly from the absence of a correlation between the two matrices behaviourally. In the future, for word sets where affective and co-occurrence-based similarities show a weak but significant correlation, it will be worthwhile to examine what remains after partialling out the effect of the respective matrices.

The visual control stimuli consisted of consonant letter strings and the auditory control stimuli of rotated spectrograms. Hence, in the visual modality, the stimuli still contain linguistic symbols but not in the auditory control stimuli. This is unlikely to affect the results, which are based on pooled analysis of auditory and visual word trials.

The sensitivity of the RSA is difficult to determine accurately as there is no groundtruth. This is the case for most functional imaging studies of cognition. The sensitivity depends on a number of factors and not only on the number of words examined. Other factors that determine the sensitivity and specificity are the number of within-subject replications per word (8 in our study), the range of similarities between the words in the word set (maximised in our study using as semiautomated procedure), the number of individuals scanned, the intertrial interval duration and the hemodynamic modelling procedure. These multiple factors together determine sensitivity and specificity.

When association-based similarities were partialled out, the representational similarity effect for co-occurrence-based similarities disappeared. This emphasises the shared information in both word association models. Co-occurrence-based models and free word association models both tap into word meaning as derived from word context. With a correlational approach the collinearity between the different measures requires caution for exclusionary explanations. Hence the findings should not be interpreted in terms of a general superiority of one type of models versus another in modelling word associations Neither is it possible to empirically ascertain whether the correlation between the co-occurrence-based matrix and the fMRI-activity-based similarity matrix reflects any correspondence in underlying computational mechanisms. The correlation between the co-occurrence-based matrix and the fMRI-activity-based similarity matrix may also be indirect via the associations between word meanings. Hence the proposal that co-occurrence-based mechanisms may play a role in coding of word meaning in lateral temporal cortex remains hypothetical at the current stage.

## CONCLUSIONS

The results of this study lead us to hypothesise that the brain uses several behavioural and distributional mechanisms to construct word meaning, in accordance with a hybrid framework of embodied and symbolic representations. While experiential information is represented in a more distributed fashion, corpus-based information correlates with patterns in a focalised region surrounding the STS. The latter relies on statistical regularities that allow for predictive coding.

## FUNDING INFORMATION

Rik Vandenberghe, Onderzoeksraad, KU Leuven (https://dx.doi.org/10.13039/501100004497), Award ID: C14/17/108. Rik Vandenberghe, Onderzoeksraad, KU Leuven (https://dx.doi.org/10.13039/501100004497), Award ID: C14/21/109. Rik Vandenberghe, Fonds Wetenschappelijk Onderzoek (https://dx.doi.org/10.13039/501100003130), Award ID: G094418N. Antonietta Gabriella Liuzzi, Fonds Wetenschappelijk Onderzoek (https://dx.doi.org/10.13039/501100003130), Award ID: 1247821N.

## AUTHOR CONTRIBUTIONS

**Antonietta Gabriella Liuzzi**: Formal analysis: Supporting; Methodology: Supporting; Writing – review & editing: Supporting. **Karen Meersmans**: Conceptualization: Lead; Data curation: Lead; Formal analysis: Lead; Investigation: Lead; Methodology: Lead; Writing – original draft: Lead; Writing – review & editing: Lead. **Gerrit Storms**: Conceptualization: Supporting; Writing – review & editing: Supporting. **Simon De Deyne**: Formal analysis: Equal; Methodology: Equal; Software: Supporting; Writing – review & editing: Supporting. **Patrick Dupont**: Methodology: Equal; Software: Equal; Writing – review & editing: Equal. **Rik Vandenberghe**: Conceptualization: Lead; Funding acquisition: Lead; Methodology: Equal; Supervision: Lead; Writing – original draft: Equal; Writing – review & editing: Lead.

## DATA AVAILABILITY STATEMENT

Data are available on https://github.com/lcn-kul/affective-similarities.

## TECHNICAL TERMS

Small World of Words: Publicly available data set obtained by means of online responses of more than 70,000 individuals to more than 12,000 words, performed for different native languages.
Representational similarity analysis (RSA): Method where the similarity between experimental stimuli (such as words) is related to the similarity between the activity patterns in response to these stimuli.
