# Supplementary material for: Independency of Coding for Affective Similarities and for Word Co-occurrences in Temporal Perisylvian Neocortex
Source: Neurobiol Lang (Camb). 2023 Apr 11;4(2):257–79. doi: 10.1162/nol_a_00095 (PMC10205158; doi:10.1162/nol_a_00095)
Supplement: Supplementary file 1 [file nol-4-2-257-s001.docx]

Supplementary Material:

Table1: Stimulus dataset. For each Dutch noun, the English translation is provided.

Supplementary figure 1. Q-Q plot of the similarities derived from spaCy, task-based associations and affective ratings. If the blue dots fall along the black reference line, the data comes from a normal distribution. These plots demonstrate the skewedness of the Small World of Words and affective similarities, justifying the use of Spearman correlation in the whole-brain RSA.


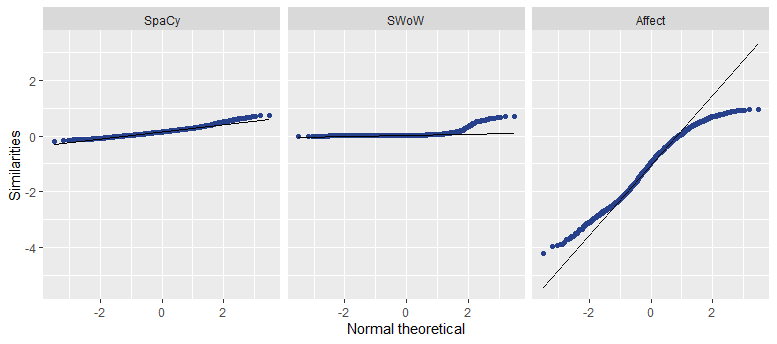


| **Table 2 \| Results of the Searchlight whole-brain Representational Similarity Analysis for co-occurrence-based similarities (spaCy) corrected for (A) phonological distance, (B) neighbourhood density, (C) taxonomy-based and (D) experiential-strength similarity.** Spearman correlation was used for all analyses. Significance was set at a whole-brain FWE-corrected threshold of p < 0.05 (with uncorrected voxelwise p < 0.001). Size: cluster size in number of voxels. FWE p: FWE-corrected p-value at the cluster-level.  *Abbreviations:* STS: Superior Temporal Sulcus. | | | | | | |
| --- | --- | --- | --- | --- | --- | --- |
| (A) Searchlight RSA results for co-occurrence-based similarities corrected for phonological distance | | | | | | |
| **Label** | **Size** | **Peak coordinates** | | | **t(21)** | **FWE p** |
| L posterior STS | 1118 | -57 | -34 | 2 | 5.51 | <0.001 |
|  |  | -27 | -61 | 29 | 5.25 |  |
|  |  | -15 | -70 | 41 | 4.91 |  |
| R posterior STS | 978 | 54 | -37 | 5 | 5.20 | <0.001 |
|  |  | 48 | -28 | 5 | 5.19 |  |
|  |  | 57 | -22 | -1 | 5.13 |  |
| (B) Searchlight RSA results for co-occurrence-based similarities corrected for neighbourhood density | | | | | | |
|  | | | | | | |
| **Label** | **Size** | **Peak coordinates** | | | **t(21)** | **FWE p** |
| Left posterior STS | 749 | -57 | -34 | 2 | 5.37 | < 0.001 |
|  |  | -54 | -13 | -4 | 4.71 |  |
|  |  | -69 | -1 | -4 | 4.48 |  |
| Right posterior STS | 973 | 54 | -37 | 5 | 5.29 | < 0.001 |
|  |  | 48 | -28 | 5 | 5.22 |  |
|  |  | 57 | -22 | -1 | 4.96 |  |
| (C) Searchlight RSA results for co-occurrence-based similarities corrected for taxonomy-based similarity (WordNet) | | | | | | |
| **Label** | **Size** | **Peak coordinates** | | | **t(21)** | **FWE p** |
|  |  |  | | |  |  |
| Left posterior STS | 789 | -57 | -34 | 2 | 6.04 | < 0.001 |
|  |  | -54 | -13 | -1 | 4.61 |  |
|  |  | -66 | -4 | -4 | 4.43 |  |
| Right posterior STS | 888 | 54 | -37 | 5 | 5.24 | < 0.001 |
|  |  | 60 | -25 | -4 | 5.10 |  |
|  |  | 51 | -28 | 2 | 4.93 |  |
| (D) Searchlight RSA results for co-occurrence similarities corrected for experiential-strength similarity (Lancaster) | | | | | | |
| **Label** | **Size** | **Peak coordinates** | | | **t(21)** | **FWE p** |
| Left posterior STS | 252 | -57 | -34 | 2 | 4.78 | < 0.015 |
|  |  | -54 | -13 | -4 | 4.16 |  |
|  |  | -66 | -4 | -4 | 3.87 |  |
| Right posterior STS | 578 | 57 | -16 | -7 | 4.91 | < 0.001 |
|  |  | 66 | -19 | -7 | 4.87 |  |
|  |  | 54 | -37 | 5 | 4.34 |  |

| **Table 3 \| Results of the Searchlight whole-brain Representational Similarity Analysis for affective similarities corrected for (A) phonological distance, (B) neighbourhood density, (C) taxonomy-based and (D) experiential-strength similarity.** Spearman correlation was used for all analyses. Significance was set at a whole-brain FWE-corrected threshold of p < 0.05 (with uncorrected voxelwise p < 0.001). Size: cluster size in number of voxels. FWE p: FWE-corrected p-value at the cluster-level.  *Abbreviations:* STS: Superior Temporal Sulcus; MTG: middle temporal gyrus; FC: frontal cortex; FG: fusiform gyrus. | | | | | | |
| --- | --- | --- | --- | --- | --- | --- |
| (A) Searchlight whole-brain RSA results for affective similarities corrected for phonological distance | | | | | | |
| **Label** | **Size** | **Peak coordinates** | | | **t(21)** | **FWE p** |
| Left and right STS, MTG, precentral gyrus and precuneus. Left middle and inferior FC. Left FG. | 13306 | -69 | -25 | 5 | 8.63 | <0.001 |
|  |  | -51 | -34 | 5 | 7.54 |  |
|  |  | -36 | -64 | -1 | 7.35 |  |
| (B) Searchlight RSA results for affective similarities s corrected for neighbourhood density | | | | | | |
| **Label** | **Size** | **Peak coordinates** | | | **t(21)** | **FWE p** |
| Left and right STS, MTG, precentral gyrus and precuneus. Left middle and inferior FC. Left FG. | 13010 | -69 | -25 | 5 | 8.58 | < 0.001 |
|  |  | -51 | -34 | 5 | 7.54 |  |
|  |  | -36 | -64 | -1 | 7.20 |  |
| (C) Searchlight RSA results for affective similarities corrected for taxonomy-based similarity (WordNet) | | | | | | |
| **Label** | **Size** | **Peak coordinates** | | | **t(21)** | **FWE p** |
| Left STS, MTG, middle and inferior FC, precentral gyrus, precuneus and FG. | 7544 | -69 | -25 | 5 | 8.75 | < 0.001 |
|  |  | -54 | -28 | 8 | 7.55 |  |
|  |  | -36 | -64 | -1 | 7.48 |  |
| Left STS, MTG, middle and inferior FC, precentral gyrus, precuneus and FG | 4326 | 57 | -19 | 2 | 6.92 | < 0.001 |
|  |  | 51 | -40 | 2 | 6.22 |  |
|  |  | 51 | -1 | 44 | 5.83 |  |
| (D) Searchlight RSA results for affective similarities corrected for experiential-strength similarity (Lancaster) | | | | | | |
| **Label** | **Size** | **Peak Coordinates** | | | **t(21)** | **FWE p** |
| Left STS, MTG, middle and inferior FC, precentral gyrus, precuneus, FG | 6680 | -69 | -25 | 5 | 9.14 | < 0.001 |
|  |  | -36 | -64 | -1 | 7.13 |  |
|  |  | -69 | -25 | -13 | 6.66 |  |
| Right STS, MTG, precuneus, supramarginal gyrus, FG | 2268 | 57 | -19 | 2 | 6.78 | < 0.001 |
|  |  | 51 | -40 | 2 | 6.23 |  |
|  |  | 60 | -43 | 11 | 5.83 |  |
| Right precentral gyrus, middle and inferior FC | 569 | 51 | -1 | 44 | 4.82 | < 0.001 |
|  |  | 18 | 2 | 50 | 4.64 |  |
|  |  | 24 | -4 | 47 | 4.58 |  |
